# Bexar County Medical Society

**Published:** 1904-02

**Authors:** J. H. Burleson

**Affiliations:** Secretary


### Bexar County Medical Society.

An unusually interesting meeting of the Bexar County Medical Society was held in San Antonio on the evening of January 7th. The newly elected president, Dr. W. B. Russ, presided. Major Charles F. Mason, U. S. A., read a paper on "The Role of Insects as Carriers of Infection," which was thoroughly enjoyed and much discussed. Dr. G. Graham Watts opened the discussion and was followed by Adolph Herff, E. Cross, F. Paschal, W. M. Wolf, T. J. Largen, B. F. Kingsley, Col. Jos. L. Girard and others. The society then unanimously adopted a resolution instructing the secretary to have 1000 copies of Dr. Mason's paper printed for general distribution.

Under the head of topics for impromptu discussion the subject, "The Treatment of Specific Endometritis," was announced, and Dr. F. E. Young was called upon to open the discussion. Following Dr. Young were Drs. E. Cross. Russell Caffery, E. M. Rabb, F. Paschal, A. Herff, J. Herff and B. F. Kingsley. The second topic was "The Value of Antitoxin in the Prevention and Treatment of Diphtheria." Dr. J. V. Spring led the discussion and he was followed by Dr. F. M. Hicks, T. E. Moore, R. Robinson, E. C. Clavin, A. O. Brown and T. T. Jackson.

The following committees were announced by the president:

Committee to Draft Resolutions of Respect to the Memory of the late Dr. H. A. West.—Drs. T. T. Jackson, A. Herff, G. G. Watts, J. H. Bell and H. D. Barnitz.

Committee to Arrange for a General Meeting of Representatives from all the Societies in the District.—Drs. J. S. Lankford, E. T. Hughes, C. M. Decker, E. Cross and S. T. Lowry.

Committee to Entertain the State and Federal Health Authorities, soon to visit San Antonio.—Drs. D. Berrey, J. S. Lankford, F. E. Young, F. Paschal and Charles F. Mason.

Committee to Investigate the Feasibility of Establishing a Medical Library in San Antonio.—Drs. M. J. Bliem, J. V. Spring and Robt. E. Moss

Committee to Secure a Supply of Antitoxin for the Benefit of Families too Poor to Purchase It.—Drs. W. L. Barker, T. E. Moore, D. Berrey, S. Burg and L. L. Shropshire.

The following new members were elected: Drs. R. L. Felts, U. S. A., R. L. Withers, G. de la Lama, G. W. Johnson and J. D. Bell. This places the membership at 88, and makes the Bexar County Medical Society the largest in the State.

There was a very large attendance.

J. H. Burleson, M. D.,
Secretary.

